# Odontogenic Fibromyxoma of Maxilla: A Rare Case Report

**DOI:** 10.1155/2013/345479

**Published:** 2013-03-04

**Authors:** G. Siva Prasad Reddy, B. Surya Kumar, Radhika Muppa, Shravan Kumar Regonda, Harish Kumar TVS

**Affiliations:** ^1^Department of Oral and Maxillofacial Surgery, Panineeya Institute of Dental Sciences, Road No. 5, Kamala Nagar, Dilsukhnagar, Hyderabad, Andhra Pradesh 500060, India; ^2^Department of Dental Surgery, Mediciti Hospital, Near Secretariate, Hyderabad, India; ^3^Department of Pedodontics, Panineeya Institute of Dental Sciences, Hyderabad, India

## Abstract

Fibromyxoma is a rare odontogenic tumour which is benign, but locally aggressive. The etiology of these tumours is unknown, but because of its limitation to the teeth bearing areas and occasional presence of odontogenic epithelial fragments within the tumour which suggest that it is of odontogenic origin. It is a slow growing painless tumour that frequently occurs in second and third decades of life. Females are more commonly affected than males. The tumour can cause gradual expansion of the cortical plates and cause loosening and displacement of teeth, although root resorption may be rare. The surgical treatment of these tumours consists of complete enucleation or radical excision. The aim of this paper is to present the rarity of a fibromyxoma of the maxilla.

## 1. Introduction

Fibromyxoma is a benign tumour of ectomesenchymal origin with or without odontogenic epithelium [1]. The term myxoma was first used by Virchow in 1863, but the term fibromyxoma was described by Dietrich et al. [2]. The tumour occurs in soft and bony tissues. Earlier, the bone myxomas were subclassified into osteogenic and odontogenic entities [3]. Myxomas can be found in heart, skin, and subcutaneous tissue and centrally in the bone, but myxomas of the jaws are encountered rarely. It accounts for only 1% to 3% of all cysts and tumors of the jaws [4]. Mandible is more frequently affected than the maxilla. They frequently occur in 2nd and 3rd decades of life. Females are more commonly affected than males. Some studies reveal a lack of sex predilection and equal frequency of the maxillary and mandibular involvements [5]. The myxomas usually appear as solitary growths, and they are extremely rare in children under the age of 12 years [6]. The size of the tumour varies, and it may reach up to 4 cm in diameter. Small myxomas are treated conservatively with curettage followed by cauterization. Larger tumours may require extensive resection, as recurrence rates as high as 25% have been reported. Fibromyxoma of the jaw appears to have a better prognosis than does fibromyxoma occurring in the long bones of the skeleton. 

## 2. Case Report

**Figure 1:**
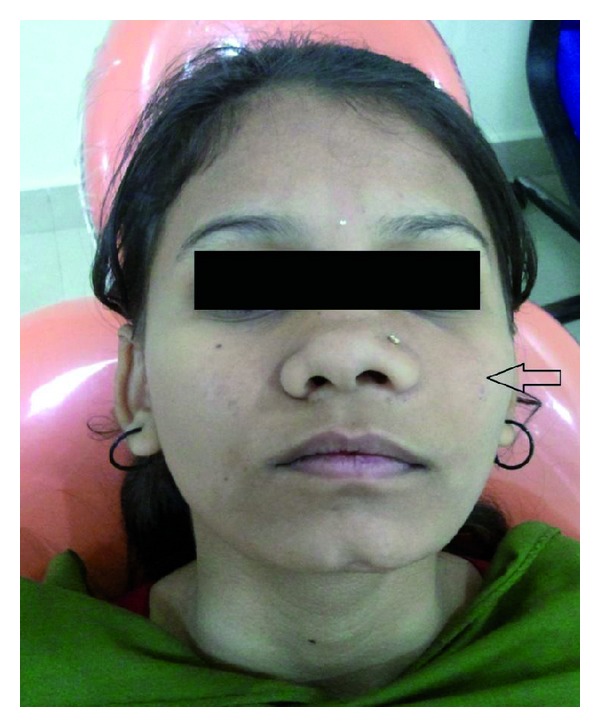
Preoperative frontal view showing swelling on left side of the face.

**Figure 2:**
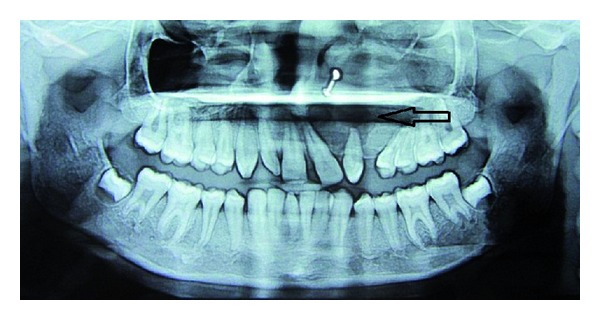
Orthopantomograph showing ill-defined mixed radiolucency in the left maxilla with displacement of the adjacent teeth.

**Figure 3:**
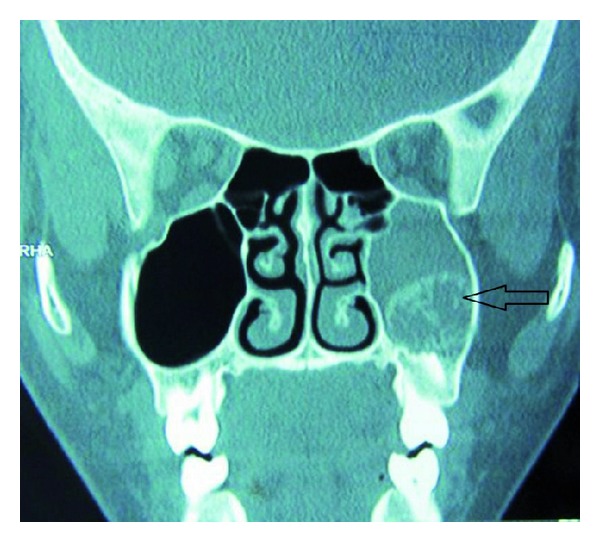
Coronal CT scan section showing the lesion completely obliterating the left maxillary sinus.

**Figure 4:**
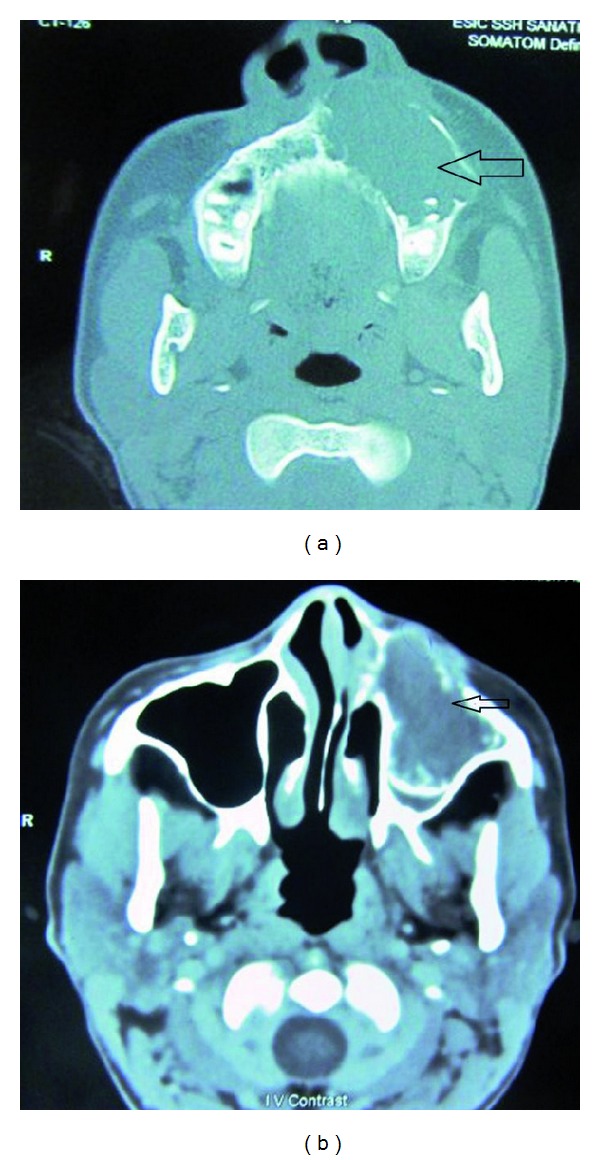
Axial CT Scan sections showing the lesion perforating the anterior wall of the antrum and its extension in to the nasal cavity.

**Figure 5:**
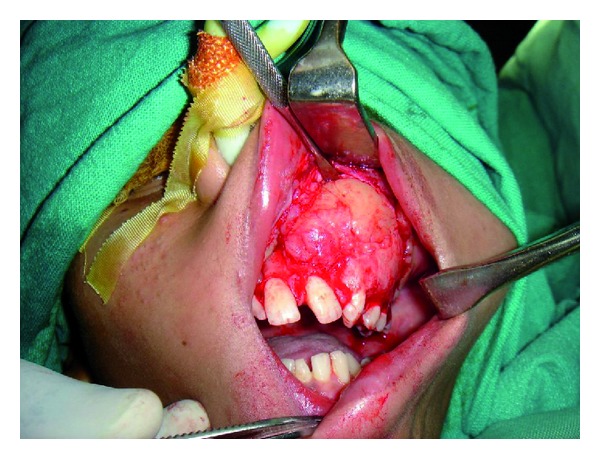
Exposure of the tumour by an intraoral approach.

**Figure 6:**
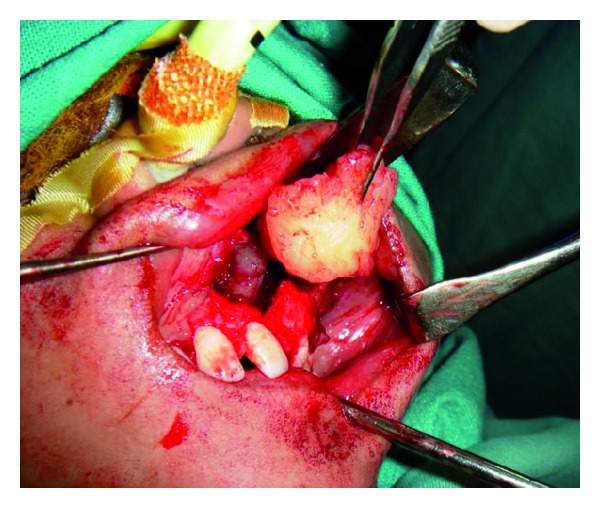
Curettage and complete removal of the lesion.

**Figure 7:**
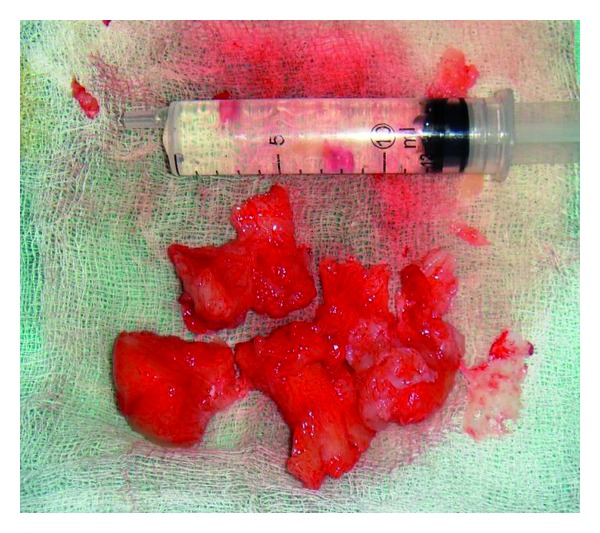
Gross appearance of the tumour mass.

**Figure 8:**
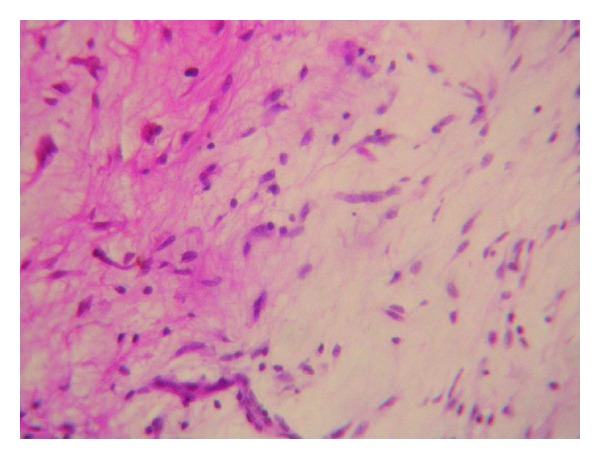
Histopathological examination showing spindle-shaped fibroblasts in loose myxoid tissue.

**Figure 9:**
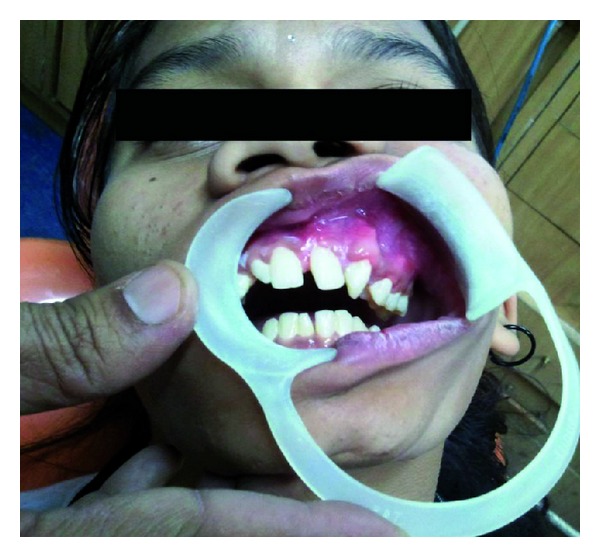
Postoperative image showing intraoral healing.

A 12-year-old female patient visited our unit with a chief complaint of swelling on left side of the face since 6 months. Extraoral examination revealed diffuse swelling over the left cheek involving lateral part of the nose and infraorbital region (Figure 1). Skin over the swelling was normal without any tenderness or surface discolouration. Intraoral examination revealed a firm to hard swelling with buccal cortical plate expansion in relation to left upper canine-premolar region. Mucosa over the swelling was normal without any draining sinuses. The palatal aspect was devoid of any such swelling, and the mucosa appeared normal. Upper left central incisor and canine teeth were tilted, and the lateral incisor was missed. Orthopantomograph revealed an ill-defined mixed radiolucency in the left maxilla with displacement of adjacent teeth (Figure 2). Computed tomography sections showed the lesion completely obliterating the left maxillary sinus with intact palatal bone and posterior wall of the sinus (Figures 3, 4(a), and 4(b)). An incisional biopsy was performed under local anesthesia, and the histopathological examination revealed the lesion as fibromyxoma. Complete curettage and enucleation of the lesion was then performed by an intraoral approach under general anesthesia (Figures 5 and 6). The enucleated tumour mass was submitted to oral and Maxillofacial Pathology Department (Figure 7). The histopathology showed spindle-shaped fibroblasts in loose myxoid tissue and confirmed the diagnosis (Figure 8). Patient came for regular followups, and there was no recurrence of the lesion with good intraoral healing (Figure 9).

## 3. Discussion

Fibromyxoma is a rare aggressive intraosseous lesion derived from embryonic mesenchymal tissue associated with odontogenesis. The maxilla and anterior region of the mandible are rarely affected. When found in the maxilla it usually behaves more aggressively than that of the mandible. It involves the zygoma, maxillary sinus, and even the orbits. 

Evidence that may support the odontogenic origin of fibromyxoma of the jaws was contributed by Thoma in 1934. He stated that the greater portion of myxomas that occur in the jaws are derived from embryonic tissues of the dental papilla, the dental follicle, or the periodontal membrane [7, 8]. Most myxomas are asymptomatic, although some patients present with progressive pain in lesions involving the maxilla and maxillary sinus, with eventual neurologic disturbances.

Radiographically, the fibromyxomas present themselves as multilocular or unilocular radiolucency with well-defined borders. Most of them are multilocular. Radiological investigations reveal homogenous radiolucencies with different appearances like honey comb, soap bubble, or tennis racket [1, 9]. Differential diagnosis for a unilocular radiolucency can be any cyst, and for a multilocular radiolucency it can be ameloblastoma, central hemangioma, and odontogenic keratocyst. An aspiration biopsy would be nonproductive.

Grossly the tumour is greyish-yellow multinodular tissue. Its consistency varies. Some portions of the tumour may be sticky, gelatinous, or semisolid, and others may be firm. The surface of the tumour is shiny and glistening.

Histologically the fusiform or stellate cells are elongated, having cytoplasmic processes stretching in various directions. It contains few cells that lie in a myxoid ground substance being elongated with spindle-shaped nuclei and having the appearance of fibrocytes. It is of diagnostic challenge to the examiners because it is difficult to differentiate from the other odontogenic tumours. Mitotic figures are rarely seen. The ultrastructural studies of an odontogenic myxoma demonstrate the presence of two basic functional types of cells: secretory and nonsecretory. The secretory cell type is the principal tumour cell and resembles the fibroblast [10]. 

The tumour is not radiosensitive, and hence surgery is the only treatment of choice. The surgical treatment of the fibromyxoma involves enucleation and curettage, radical excision, en bloc resection. The avoidance of recurrence is strongly related to the complete resection of the lesion. Thomas has stated that recurrence of fibromyxoma is uncommon if enucleation is complete. Myxomas or fibromyxomas show a recurrence rate between 25% and 43%. Harder et al. stated that rate of recurrence varies widely as does the choice of treatment. Their study supports conservative surgery as the appropriate treatment for odontogenic myxomas, as they have found no evidence of malignant change or increasingly aggressive behaviour in any of the recurrences [8].

Chen et al. proposed conservative treatment as the preferred method for excision with extensive resection reserved for large tumors. In the long bones the tumour is frequently malignant and tends to recur with great frequency after removal, but fibromyxomas of the jaws appear to have a better prognosis.

## 4. Conclusion

In conclusion, fibromyxomas of the jaws are rare but not uncommon. The prime treatment considerations include the age of the patient and recurrence of the lesion. The tumour is not radiosensitive, and surgery is the only choice of treatment. In this young unmarried girl as the extent of lesion is confined to the maxillary sinus, we have opted for an intraoral conservative surgical approach.
